# Functional Characterization of Cholinergic Receptors in Melanoma Cells

**DOI:** 10.3390/cancers12113141

**Published:** 2020-10-27

**Authors:** Anna Maria Lucianò, Ada Maria Tata

**Affiliations:** 1 Department of Biology and Biotechnologies Charles Darwin, Sapienza University of Rome, 00185 Rome, Italy; anna.luciano94@gmail.com; 2 Research Centre of Neurobiology Daniel Bovet, Sapienza University of Rome, 00185 Rome, Italy

**Keywords:** cholinergic system, acetylcholine, muscarinic receptors, nicotinic receptors, melanoma, cancer

## Abstract

**Simple Summary:**

Interest in the involvement of the cholinergic system at the non-neuronal level emerged in recent years, thereby allowing to identify different physiological and pathological processes that acetylcholine and its receptors modulate in different tissues. The cholinergic system can also take part in the activation and maintenance of pathological processes such as neurodegenerative diseases and cancer. This review summarizes studies concerning the involvement of the cholinergic system in different tumor isotypes, with a special focus on melanoma, the most lethal form of skin cancer.

**Abstract:**

In the last two decades, the scientific community has come to terms with the importance of non-neural acetylcholine in light of its multiple biological and pathological functions within and outside the nervous system. Apart from its well-known physiological role both in the central and peripheral nervous systems, in the autonomic nervous system, and in the neuromuscular junction, the expression of the acetylcholine receptors has been detected in different peripheral organs. This evidence has contributed to highlight new roles for acetylcholine in various biological processes, (e.g., cell viability, proliferation, differentiation, migration, secretion). In addition, growing evidence in recent years has also demonstrated new roles for acetylcholine and its receptors in cancer, where they are involved in the modulation of cell proliferation, apoptosis, angiogenesis, and epithelial mesenchymal transition. In this review, we describe the functional characterization of acetylcholine receptors in different tumor types, placing attention on melanoma. The latest set of data accessible through literature, albeit limited, highlights how cholinergic receptors both of muscarinic and nicotinic type can play a relevant role in the migratory processes of melanoma cells, suggesting their possible involvement in invasion and metastasis.

## 1. Introduction

### Non-Neuronal Acetylcholine

Acetylcholine (ACh) was the first molecule identified as a neurotransmitter operating through two subgroups of receptors, namely muscarinic receptors (mAChRs) and nicotinic receptors (nAChRs) (Table 1 and Table 2). The presence of this neurotransmitter in different primitive organisms (e.g., bacteria, protozoa, fungi, algae) highlights its relevance not only as mediator in the nervous system, but also as a non-neuronal signaling molecule involved in the regulation of basic cell functions like proliferation, differentiation, cell–cell contact, and secretion, thus making ACh phylogenetically, the oldest signaling molecule [1,2,3,4,5].

The importance of ACh as a modulator of different biological processes and the concept itself of non-neuronal acetylcholine function, has been growing since the early 1970s, capitalizing on the discoveries of functional cholinergic receptors in non-neural cells [6]. In particular, the muscarinic receptors have been identified in various organs such as retina [7], lymphocytes [8], spleen [9], adrenal gland [10] and stomach [11]. In the same years, the expression of nicotinic receptors was first documented outside of the nervous system [12]. Locating acetylcholine receptors in different districts has moved in synchrony with identifying the roles played by the latter at non-neuronal level. In this context, it was demonstrated during sea urchin embryogenesis how ACh acts as a morphogen, controlling cell migration and regulating basic cell functions including growth, survival, differentiation, and apoptosis [13,14]. It is also relevant to note that in several tissues, AChRs are not just a target of ACh-deriving from nervous system innervation. In fact, several non-neuronal cells appear able to synthesize and release ACh autonomously, highlighting possible autocrine/paracrine effects mediated by non-neuronal ACh [15,16]. The involvement of ACh in non-neuronal functions was also supported by the demonstration of the cholinergic modulation of the inflammatory states. Several authors reported lymphocytes, dendritic cells, and macrophages clearly express cholinergic components sufficient to constitute their own cholinergic system, and that immune cells and peritoneal macrophages express all five muscarinic receptors and several nAChR subunits. Furthermore, during immunological reaction, stimulated T cells and dendritic cells have the ability to synthesize and release ACh, which in turns acts in an autocrine–paracrine manner on muscarinic or nicotinic receptors expressed in the immune cells, modulating the immune response and inflammatory processes. The same evidence indicates that ACh is able to inhibit the release of tumor necrosis factor and IL1 from macrophages via the activation of nicotinic receptors, thus highlighting the existence of a cholinergic anti-inflammatory pathway [17,18,19]. Lastly, the microglial cells in the central nervous system are also able to respond to ACh stimulation, inhibiting the release of TNF-α. These data also suggest the existence of a “brain cholinergic anti-inflammatory pathway,” mediated by α7 nicotinic receptors, which may become of interest in the treatment of the neuroinflammation, typically established by neurodegenerative processes [20,21]. Similarly, in the tumor cells, the ACh can be synthesized and released locally in the tumor bulk and actively participate in tumorigenesis processes [22,23]. Specifically for the epithelial cells, the first discovery of acetylcholine production in the epithelial cells came in 1983, thanks to the study conducted by Mark [24] on salivary glands of rats, which continued to produce large amounts of ACh despite denervation. Subsequently, it has been proved that human epidermal keratinocytes possess cholinergic enzymes, which synthesize and degrade acetylcholine, and express both nicotinic and muscarinic classes of cholinergic receptors on their cell surfaces. Consequent to ACh release, these receptors are able to initiate a cell response [25]. Furthermore, epidermal and gingival keratinocytes express the M1–M5 and M2–M5 mAChR subtypes, respectively, as well as the α3, α5, α7, α9, and β2 nAChR subunits [26,27,28]. ACh synthesized by and released from keratinocytes acts on both mAChRs and nAChRs in an autocrine and/or paracrine manner, modulating basic cell functions in the skin, including keratinocyte proliferation, differentiation, adhesion and migration, as well as epidermal barrier formation. Similarly, ACh is required to sustain viability also of the keratinocytes in vitro [29]. Chernyavsky and collaborators demonstrated that keratinocyte migration is modulated by distinct muscarinic acetylcholine receptor subtypes. Additional studies showed that M4 receptor increased the expression of “migratory” integrins, whereas M3 receptor upregulated “sedentary” integrins, demonstrating that the M3 muscarinic receptor is able to inhibit the migration via the guanylyl cyclase–cyclic GMP–protein kinase G signaling pathway [30]. Based on these data, it appears evident that the role of ACh should be largely reconsidered, both at physiological and pathological level. A deeper knowledge of the mechanisms through which ACh mediates its effects may open to new perspectives for the development of therapeutic protocols for different pathologies.

## 2. Cholinergic Receptor Subtypes: Structure and Function

### 2.1. Nicotinic Acetylcholine Receptors

The nAChRs are fast ionotropic cationic acetylcholine receptors that mediate the fast-synaptic transmission, whose activation is extremely quick (msec to sub-msec) [31,32]. They are pentameric transmembrane protein complexes, formed by five receptor subunits that enclose a central ion channel [33]. At present, several subunits have been identified and characterized, comprising 10 α subunits (from α1 to α10), 4 β subunits (from β1 to β4), 1 δ, and 1 ε or 1γ subunits [17]. Among all nAChR subunits, the α8 subunit is the only one not present in human cells [34]. In neurons, the nAChRs can be homopentamers formed by α7, α8, and α9 subunits or heteropentamers formed by combination of α and β subunits. In muscle, functional heteropentamer consists of two α subunits and one of each β, γ, and δ subunits. All the nAChRs show permeability to various cations, such as Na^+^, Ca^2+^ and K^+^, with α7 nAChR displaying the highest permeability for the Ca^2+^. In fact, the binding of nAChRs to acetylcholine or nAChRs agonists leads to membrane depolarization and opening of voltage-gated ion channels, resulting in ion influx and efflux [31]. The channel activity is the first step in the activation of these receptors, which leads subsequently to a number of other intracellular events and various downstream signaling pathways, such as activation of mitogen-activated protein kinase (MAPK), protein kinase C, and vascular endothelial growth factor, depending on the nAChR subtype and the cell-type involved [35].

#### Involvement of Nicotinic Acetylcholine Receptors in Cancer

As previously described, the nicotinic acetylcholine receptors are expressed not only in the neuronal system, but also in numerous non-neuronal tissues such as skin, pancreas, and lung, thus suggesting a role in other biological processes in addition to synaptic transmission. Specifically, these receptors were found to play a role in the regulation of cellular processes such as cell proliferation and cell death [36].

It is known that a dysregulation of nAChRs is associated with neurological disorders such as Alzheimer, Parkinson’s disease, and schizophrenia [37]. Moreover, studies performed in lung cancer highlighted that the activation of the nAChRs pathways may affect cell proliferation, apoptosis, angiogenesis, and epithelia mesenchymal transition (EMT), supporting a pro-invasive phenotype [38]. Crucial for this discussion is that cancer cells can synthesize ACh, promoting tumorigenesis through AChRs in the absence of nicotine or other agonists. The release of acetylcholine was first demonstrated in small-cell lung cancer (SCLC) and expression of choline acetyltrasferase was found in different SCLC cell lines. Acetylcholine released was inhibited by vesicular ACh transporter (VAChT) inhibitor, vesamicol, in a dose-dependent manner, suggesting a vesicular ACh release also in non-neuronal tissues [39]. Similar findings were also reported in colon and gastric cancer models. In particular, in gastric cancer, acetylcholine production promoted cell growth in a dose-dependent manner, and acetylcholine-stimulated cell proliferation was abolished by AChRs antagonists. Similarly, colon cancer cells can release the self-produced acetylcholine, which promoted cell viability in an autocrine manner [40]. The pathways activated in cancer processes by α7-nAChR activation-nicotine mediated are generally Ras/ERK/MAPK and JAK2/STAT/-PI3K pathways, leading to cancer cell proliferation and migration as demonstrated in lung cancer cells [41,42].

### 2.2. Muscarinic Acetylcholine Receptors

The other class of acetylcholine receptors is represented by muscarinic receptors, which belong to the class of heptaelical G protein-coupled receptors and consist of five subtypes (M1–M5). Like all the G protein-coupled receptors, they share a structure composed of seven transmembrane helices (TM1–TM7) connected by three intracellular (i1–i3) and three extracellular (e1–e3) loops. The receptor activation occurs always via coupling to heterotrimeric guanine nucleotide-binding proteins and subsequent kinases activation or ion channel activity regulation [43,44].

The odd muscarinic receptors, M1, M3, and M5, are coupled to the Gq11 protein, which stimulates the IP3 hydrolysis and the intracellular calcium mobilization. They also modulate phospholipase A2 and phospholipase D in different cell lines and primary cultures [45,46,47]. M2 and M4 receptors are coupled with the Gi protein, which inhibits adenylate cyclase activity, thus reducing cAMP intracellular levels. They are also able to modulate the activity of K^+^ channels. Muscarinic receptor subtypes can also activate small G proteins, such as Rho GTPase and recruit new effectors including IP3K and MAPK/ERK kinases. These last signaling pathways appear to play important roles in the control of cell growth and proliferation, as suggested by studies on glial cell primary cultures [48,49,50,51,52,53,54]. Moreover these receptors play a strategic role in the control of relevant physiological functions [55,56]. Generally, M2Rs in central nervous system (CNS) are inhibitory auto- or heteroreceptors, regulating several processes such as thermoregulation, cognitive functions, behavioral flexibility, and memory [57,58,59,60]. In the last years, the involvement of these receptors has also been investigated in different neurological pathologies such as Alzheimer’s and Parkinson’s diseases, which are characterized by significant reduction of M1 mAChRs expression [59,60]. The M3 muscarinic receptor (M3R) is located in different districts in the body, e.g., smooth muscles, endocrine and exocrine glands, lungs, pancreas, and brain. Their localization in the brain is relevant in hypothalamus and brainstem [61]. These receptors are highly expressed on pancreatic beta cells and are critical regulators of glucose homeostasis by modulating insulin secretion [62]. M3R mediates the ACh functions in the central and peripheral nervous system, with various relevant physiological functions. However, peripheral M3Rs are critical in mediating ACh functions in smooth muscle and glandular tissues [63,64,65]. The M4R is primarily located in the CNS and belongs to the family of Gi/o protein-coupled receptors [66,67]. M4R activation through adenylate cyclase inhibition, and potassium channel regulation, acts as inhibitory autoreceptor [68,69]. Dysregulation of M4R may cause different types of mental disorders such as schizophrenia, and neurodegenerative disorders, such as Parkinson’s and Alzheimer’s diseases [60,68]. The last muscarinic subtype, M5 receptor (M5R), belongs to the Gq protein-coupled receptor family [70]. The M5R is expressed in terminals of dopaminergic neurons. It is the only muscarinic receptor located in the ventral tegmental area (VTA), where it regulates the dopamine and glutamate release from midbrain projections [71,72,73,74,75]. It has also been proved via M5R-deficient mouse that the activation of M5R causes reduction of cerebral vascular tone, demonstrating the ability of AChRs to mediate the cerebral vessel dilation [76,77,78].

#### Involvement of Muscarinic Acetylcholine Receptors in Cancer

The distribution of all mAChRs in different tissues, such as nervous system [79], gastrointestinal tract [80], urinary bladder [81,82], heart [83], lung [84], vessels [85], and smooth muscle [86], are largely documented. Considering their involvement in different physiological processes, the alterations of their distribution or function is frequently associated with various pathologies including cancer. In particular, evidence produced in recent years has indicated a dysregulation of mAChRs in various types of human malignancies including breast, colon, prostate, and brain cancers (Table 3) [21,87,88,89,90].

The M1 receptor is involved in the progression of the prostate cancer, where it regulated cell migration and invasion through hedgehog signaling regulation [91,92,93,94].

An inhibitory effect on tumor progression was demonstrated for M2 receptor subtype. In particular in human glioblastoma, the M2 receptor stimulation by the preferential orthosteric agonist arecaidine propargyl ester, was reported to inhibit cell proliferation and survival in stable cell lines and in glioblastoma cancer stem cells isolated from human biopsies [92,93,94,95,96,97,98]. The same effect has been described in neuroblastoma cell lines [99], in urothelial bladder cancer cells [100], and in breast cancer [101].

Several data were reported on the role of the M3 muscarinic receptor in promoting tumor cell growth, invasion, migration and angiogenesis in different tumor types such as lung, breast, ovarian, and brain cancers [107]. In particular, data on lung cancer demonstrated a direct correlation between lung cancer metastasis/poor prognosis and the levels of M3 receptor [108]. Interestingly, research by Hua and collaborators proved that R2HBJJ, a novel mAChRs antagonist, can block the cholinergic autocrine loop in non-small-cell lung cancer, antagonizing the M3 receptors [109]. M3R is also involved in growth and progression of several tumors when co-expressed with epidermal growth factor receptor (EGFR) [92,93,94]. The activation of M3R results in transactivation of EGFR, thus promoting cell proliferation and inducing phosphorylation of ERK1/2 and AKT; conversely EGFR inhibition arrests the acetylcholine-induced gastric cancer proliferation [110,111,112]. In ovarian and breast cancers, the expression of M3R promotes cell viability via ERK1/2 activation; in fact, its expression is directly correlated with poor prognosis [102,103,104,105]. To date, there are limited data on M4 and M5 receptors in cancer. M4R has been described to be involved in the migration of oral cancer cells through the downstream signaling effectors, including SFKs and ERK1/2 [102]. Conversely for M5R, no data are at least available. Probably the lack of information concerning the role of M5R in cancer may in part depend on the absence of selective pharmacological ligands able to activate or block the activity of this receptor.

## 3. Melanoma

In 2012, 67,753 people were diagnosed with melanoma and 9251 individuals died from this type of tumor [103]. At the start of 21st century, melanoma remains a potentially fatal malignancy. Deaths from melanoma represent more than 75% of all skin cancer deaths [104,105,106,113]. Although the incidence of melanoma is low, as it represents only 1% of all malignant skin tumors, malignant cutaneous melanoma still represents the most aggressive and deadly form of skin cancers, affecting mainly the Caucasian population. Moreover, once metastatic, the prognosis is usually rather poor [105]. Fortunately, the increase of the molecular knowledge concerning the development of this type of cancer is allowing enormous progress towards the improvement of new targets and immunotherapies.

Melanoma arises from genetic mutations in melanocytes, the cells producing the cutaneous pigment, which can be found in the skin, eye, inner ear, and leptomeninges [114,115].

In relation to clinical and histological features, melanoma can be divided into three main subtypes: superficial spreading melanoma (SSM), nodular melanoma (NMM), and lentigo malignant melanoma (LMM) [114].

Superficial spreading melanoma is the most common type that accounts for 70% of the cases approximately. SSM may arise de novo or in association with a nevus [114]. Nodular melanoma accounts for 5% of melanomas and is more common in males than females. NMMs are often ulcerated. It does not have a radial growth phase, but only a vertical growth phase correlated with more rapid growth and higher rate of metastasis. Histologically, NMM is characterized by a predominance of dermal invasive tumor cells [114]. Lentigo malignant melanoma accounts for between 4% and 15% of cutaneous melanomas and, unlike NMM and SSM, correlates with long-term sun exposure and increasing age. LMM may evolve for decades before invading the papillary dermis. Histologically, it is characterized by a proliferation of cells that are in the basal layers of the epidermis [114]. Over the past years, several therapies have been approved by the US Food and Drug Administration (FDA) for melanoma treatment [116,117,118]. Depending on the features of the tumor (e.g., location, stage, genetic profile), the therapeutic protocol may require surgical resection, chemotherapy, radiotherapy, photodynamic therapy (PDT), and immunotherapy. For patients with stage I–IIIB melanoma, surgery is the primary treatment [118]. At present, there are two kinds of limitations in melanoma therapy. The first one is associated with adverse events, which are commonly linked to toxicity at the level of the skin or gastrointestinal tract, as well as immune reactions. The second one could be a reduced efficiency, which can occur due to resistance to chemo- or intra-lesion therapies [119]. Recently, new therapeutic targets have emerged from studies of the genetic profile of melanocytes and from the identification of molecular factors involved in the pathogenesis of the malignant transformation of the melanocytic cells [120,121,122]. In this context, it is critical to further investigate how those mechanisms may contribute to melanoma pathogenesis and progression. Hereafter, we summarize studies reporting the role of the ACh and cholinergic receptors in melanoma (Table 4), in order to better define the molecular mechanisms influencing melanoma pathobiology and to identify new possible targets for the treatment of this neoplasia.

## 4. Acetylcholine Receptors in Melanoma

### 4.1. The Nicotinic Receptors in Melanoma

The nicotinic receptors have been studied in melanoma cells, highlighting the presence of multiple subunits such as α5/α9 / α3/ β4 [123,124,126,127]. Alpha-5 nicotinic receptors (α5-nAChR), one of the homopentameric nicotinic receptors, have been identified in different forms of solid tumors. Performing an immunocytochemical analysis, it has been demonstrated that the expression of α5-nAChR is significantly increased in human melanoma tissue and melanoma cell lines, compared with normal human skin tissue [123]. The mechanism of action has been identified very recently; via the knockdown of the α5-nAChR expression in melanoma cells, it has been demonstrated that this receptor positively modulates cell proliferation, migration, and invasion. Moreover, the silencing of α5-nAChR has also demonstrated that PI3K-AKT and ERK1 are the signaling pathways activated downstream from the α5 receptors activation. Interestingly, a cross-interaction between α5 and Notch-1 pathways has also been reported. In particular, in absence of α5 receptors, the expression of Notch-1 and Hes1, the main gene downstream Notch activation, was significantly downregulated [124]. These data suggest that α5 nAChR may promote melanoma cell proliferation and metastasis through activation of AKT pathway—Notch1 mediated [124]. In addition, α9-nAChR has proven to be largely involved in promoting cancer progression both in vitro and in vivo [126,127]. In agreement with that observed in the breast cancer, α9-nAChR was also studied in melanoma. Similar to that observed in the case of α5 receptors, α9-nAChR expression was also expressed at higher levels in melanoma cells than in melanocytes. Interestingly, a first association of α9-nAChR expression with clinical-pathological features of patients affected by melanoma (e.g., clinical staging, lymph node status) was recently described [126]. In comparison with a proliferative phenotype, the mRNA levels for α9-subunit were higher in melanoma cells with the invasive phenotype than in those with the proliferative phenotype; this result was confirmed by comparing a metastatic patient group with a primary patient group. The α9-nAChR overexpression in melanoma cells has also demonstrated an increase in cell proliferation and migration; conversely, the suppression of α9-nAChR expression or activity significantly inhibited these events. Taken together, these findings suggest that α9-nAChR expression is not only relevant to melanoma growth and metastasis, but it represents a potentially unfavorable prognostic factor in melanoma patients [126].

Microarray studies and database analysis have suggested in melanoma a co-expressed upregulation of α9-nAChR and PD-L1, a protein overexpressed in melanoma cells and involved in in the regulation of EMT, cancer stemness, tumor development, metastasis formation, and resistance to therapy [127]. The silencing of α9-nAChR and overexpression studies have demonstrated a tight correlation between α9 and PD-L1 expression, suggesting that α9-nAChR may promote melanoma migration through the regulation of PD-L1 [126,127].

Albeit α9-nAChR receptor was not indicated as a prognostic factor for melanoma, different missense mutations causing an altered function of this receptor have been identified. At least 82 different mutations (in particular, 62 are nonsense mutations) have been described in various kinds of melanoma tumors (see www.cBioPortal.org).

### 4.2. Muscarinic Receptors in Melanoma

The muscarinic receptors are expressed during embryonic development by migrating neural crest cells, some of which are precursors of melanocytes [128]. Once the melanocytes differentiate, they lose the expression of the mAChRs [129] and, in the skin, only the keratinocytes maintain the expression of these receptors [29]. Interestingly, the expression of mAChRs has been demonstrated in human melanomas [129], suggesting that the tumorigenesis processes in melanoma could require the reactivated expression of these receptors.

Immunolocalization data obtained in melanoma cell lines demonstrated the presence of cholinergic markers such as choline acetyltransferase (ChAT) and acetylcholinesterase (AChE) (ACh biosynthetic and degradative enzymes, respectively), demonstrating that melanoma cells may synthesize and degrade ACh in an autonomous manner. Moreover, M3R expression at protein level was evidenced by the use of a specific antibody for M3 receptor. In addition, the presence of mRNA for M3 and M5 subtypes was reported, indicating that melanoma cells express mainly M3 subtype and probably also M5 receptor [129].

Calcium is the second messenger associated with the transduction pathway downstream from the cholinergic activation of the M1, M3, and M5 receptor subtypes. In melanoma cells, fluorometric measurements with Fura-2 have shown that in the presence of the ACh mimetic carbachol (CCh), there was an increase of intracellular Ca^2+^ concentration, indicating the functional activity of these receptors [125].

A chemotaxis measurement assay was also performed on these cells using the Boyden chamber. The melanoma cells were placed in the upper chamber in the absence or in the presence of carbachol and/or atropine (selective muscarinic antagonist), while the chemotactic agent fibronectin was placed in the lower chamber. The time taken by the cells to migrate from the upper to the lower chamber was measured as a chemotactic power of the agent used. It was observed that the addition of carbachol in the upper chamberm increased chemotaxis by 30%, while atropine inhibited it [130]. The migration of these cells was also observed in the presence of CCh or ACh using digital video microscopy; in this condition over a defined time interval, 30% of the cells showed cell body contraction and cell process retraction of more than 5 μm. Once again, the administration of atropine inhibited this contraction and retraction, indicating that the effects observed were mediated by muscarinic receptors [130].

All these evidences clearly suggest that the cholinergic activation of mAChRs on melanoma cells modulates cell migration and that, in general, their expression may be required to control invasion and metastasis formation. This may prompt the potential use of M3 antagonists to inhibit melanoma growth and metastasis, albeit this remains to be further determined. Although the M3 muscarinic receptor subtype function has been well characterized, the presence and role of other muscarinic receptor subtypes have not been thoroughly investigated in melanoma. Whilst a faint expression of M5 subtype has been described in the melanoma cells, its function remains at least unknown [130].

## 5. Conclusions

The data presented in this review clearly indicate the relevant roles played by cholinergic receptors in the cancer [21]. Comprehensive data have been reported in relation to different tumor types, such as colon and urothelial cancers [100], glioblastoma [92,93,94,95,96,97,98], neuroblastoma [99], and breast cancer [101]. Unfortunately, studies reporting the role of cholinergic receptors in melanoma are, at this stage, still limited. However, the effects mediated by M3/M5 muscarinic receptors and α5/α9 nicotinic receptors in different melanoma cell lines suggest that these receptors are involved in the modulation of cell proliferation and migration. In particular, the cell migration mediated by cholinergic receptor appears to be a function that melanoma cells recover from embryogenesis, considering that the precursors of melanocytes use the cholinergic stimuli to modulate their migration [125,128,129,130]. Although the knowledge on cholinergic system functions should be further investigated, in particular for melanoma cells obtained from human biopsies, it appears evident that acetylcholine and its receptors play an important role in tumor processes, whose better understanding could help to identify new and interesting therapeutic tools for different tumors, including melanoma.

## Figures and Tables

**Table 1:** Acetylcholine nicotinic receptors.

Types	Class	Subunits	Structure	Localization	Signaling Transduction
Nicotinic Receptors	Ionotropic	10 α subunits (α1- α10), 4 β subunits (β1 -β4), 1 δ, 1 ε, 1γ subunits	Pentameric transmembrane protein complex	Neurons, glia, ganglia, interneurons, motor endplate, immune cells	Voltage-gated ion channels, metabotropic signals (MAPK, PKC)

**Table 2:** Acetylcholine muscarinic receptors.

Subtype	Signal Transduction	Localization	Second Messengers	Function
M1	Gq, Gs, Gi	Brain, autonomic ganglia, secretory glands	IP3/DAG, NO	slow EPSP and decreased K+ conductance
M2	Gi, G0	Brain, heart, sympathetic ganglia, lung	cAMP (↓)	reduce heart rate, control contractile response, thermoregulation, cognitive functions
M3	Gi, Gq	Brain, secretory glands, smooth muscles	IP3/DAG, NO	activation of phospholipase C, phosphoinositide breakdown, and inhibition of calcium-regulated potassium channels
M4	Gi, Go	Brain, lung	cAMP (↓)	adenylate cyclase inhibition and potassium channel regulation, acts as inhibitory autoreceptor
M5	Gq	Midbrain, the ventral tegmental area (VTA)	IP3/DAG, NO	phospholipid turnover, decrease of cyclic AMP levels and downregulation of the activity of protein kinase A (PKA)

**Table 3:** Muscarinic receptors involved in cancer.

Subtype	Type of Cancer	Function	Signaling Pathways	References
M1	Prostate Cancer	Tumor cell migration and invasion	Hedgehog	[91,92,93,94]
M2	Glioblastoma, Neuroblastoma, Breast Cancer, Urothelial Bladder Cancer	Inhibition of cell proliferation, survival, migration, and chemoresistance	Notch1/EGFR	[95,96,97,98,99,100,101]
M3	Colon Cancer, Ovarian Cancer, Breast Cancer	Growth and progression of tumor, poor prognosis	ERK1/2 and AKT; EGFR	[102,103,104,105]
M4	Oral cancer	Induction of cell migration	SFKs and ERK1/2	[106]

**Table 4:** Receptor subtypes involved in melanoma progression.

Receptor Subtype	Cell Lines	Pathway Involved	Function	Reference
α5	M14 and A375	AKT/Notch-Hes ERK1/2	Cell proliferation Cell migration	[123]
α9	A375, A2058, and MDA-MB 435	STAT3 PD-L1	Cell proliferation Cell migration	[124]
M3	SK-Mel 28	Not investigated	Positive modulation of migration	[125]
